# Supplementary material for: Farmers’ adoption of soil and water conservation practices: The case of Lege-Lafto Watershed, Dessie Zuria District, South Wollo, Ethiopia
Source: PLoS One. 2022 Apr 11;17(4):e0265071. doi: 10.1371/journal.pone.0265071 (PMC9000098; doi:10.1371/journal.pone.0265071)
Supplement: Attachment — Submitted filename: Comments on Manuscript PONE-D-21-19431.docx [file pone.0265071.s002.docx]

**PONE-D-21-19431:** ***Farmers’ adoption of soil and water conservation practices: The case of Lege-Lafto Watershed, Dessie Zuria District, South Wollo, Ethiopia***

The Ethiopian highlands are important for smallholder farming communities but land degradation through soil erosion poses a major challenge for the farmers. The authors assessed adoption drivers of soil and water conservation technologies that are adapted to the conditions of the highlands. The findings of this study can potentially contribute towards efforts by the Ethiopian government to tackle land degradation through programs such as Soil and Land Management (SLM), and Agriculture Growth Program (AGP). However, in its present form, the manuscript is not publishable, and it requires major revision before it can be considered again.

**Major issues:**

1. The Introduction and Methodology sections are clear, but the Discussion of key results is very weak. Results need to be discussed in a way that brings out the ‘big picture’ implications of the key findings. Reference to literature from within and outside Ethiopia needs to be improved.

2. The Results and Discussion section has numerous grammatical errors. The authors are advised to seek editing assistance from a native English speaker.

**Minor issues:**

1. The document requires line numbers in order to facilitate easier review process

2. Avoid using words that already appear in the title as key words.

3. Introduction section

- Do not start sentences with ‘And’
- Change tone ha^-1^ yr^-1^ to t ha^-1^ yr^-1^
- Farmers are mobilised through food-for-work programs, not through food & work programs.
- Once the abbreviation for ‘Soil and Water Conservation’ has been defined in the Introduction, then use SWC throughout the rest of the document. There are instances where even both are used in the same sentence, avoid that.

4. Materials & Methods section

- Scientific names of crops should be accompanied by the naming authorities
- Clarify how the selection of each kebele in the different watershed positions was done. Was it random or systematic?
- Replace ‘plot of land’ by ‘land’
- In the text, be consistent with the refencing style, either numbers or author name(s) followed by year. Check the journal requirement.
- The different levels of the sub titles should be clear.

Results

- Tables are missing
- Avoid putting table titles between paragraphs
