# Supplementary material for: Farmers’ adoption of soil and water conservation practices: The case of Lege-Lafto Watershed, Dessie Zuria District, South Wollo, Ethiopia
Source: PLoS One. 2022 Apr 11;17(4):e0265071. doi: 10.1371/journal.pone.0265071 (PMC9000098; doi:10.1371/journal.pone.0265071)
Supplement: Attachment — Submitted filename: PONE-D-21-19431_second review.docx [file pone.0265071.s004.docx]

**Second review of**

**Farmers’ adoption of soil and water conservation practices: The case of Lege-Lafto**

**Watershed, Dessie Zuria District, South Wollo, Ethiopia**

**PONE-D-21-19431**

The study authors have made some improvements to the manuscript. A few of my earlier comments were addressed, but several comments were not adequately addressed as described below.

- The revised version has been professionally edited and this is much appreciated. However, some grammatical errors remain. Further professional editing may be needed.
- The Introduction was slightly revised, but the authors did not address my suggestions to (a) explicitly state the research gaps that the current study addresses and (b) explicitly state the research questions to be studied or hypotheses to be tested. And I’m still not clear on what it is about South Wollo that makes it particularly interesting as a case study.
- I maintain that the analyses have not been performed rigorously. A better approach (vs. the current single equation logit model) is to model jointly the decisions to adopt different agricultural technologies, as is now common practice in the agricultural technology adoption literature (e.g., Kassie et al. 2013; Ward et al., 2018). These studies show that by analyzing the complementarity and substitutability of agricultural practices the drivers of adoption and overall uptake are better understood. It is recommended that the authors review the study of Teklewold et al. (2013) who modeled the adoption of sustainable agricultural practices by Ethiopian maize farmers as a joint decision and incorporated the extent of such adoption (number of practices adopted). The authors should consider using this modeling approach.
- The authors have addressed my comment to include explanations for why the explanatory variables are included in the model, by citing previous empirical work. Thank you.
- I appreciate that the authors have tried to make better use of the qualitative data that were collected through focus group discussions (FGDs) and in-depth interviews. However, I see only one quote was added. And the FGD and KII results are only mentioned in one other part of the paper (lines 367-372). The authors should add several more quotes and summarize some of the other qualitative results at appropriate points in the paper to complement the quantitative results. That would then support the authors claim that the study is mixed methods.
- The authors have improved the Results section by weaving in a few results of related studies and discussing how the findings of the present study agree or disagree with previous work and offering plausible explanations. Thank you.
- The authors have not adequately highlighted the implications of the study findings for policy and practice. I suggest this be done in the next round of revisions.
